# Be Kind to Your Behind: A Systematic Review of the Habitual Use of Bidets in Benign Perianal Disease

**DOI:** 10.1155/2022/1633965

**Published:** 2022-05-31

**Authors:** Zarrukh Baig, Nawaf Abu-Omar, Michael Harington, Dilip Gill, David Nathan Ginther

**Affiliations:** Department of Surgery, University of Saskatchewan, Saskatoon, Canada

## Abstract

**Background:**

Benign perianal disease carries significant morbidity and financial burden on the healthcare system. Given that sitz baths are recommended as a treatment modality, we considered whether using a continuous stream of water, in the form of a bidet, offers a convenient and effective alternative. Bidet use is the predominant form of perianal hygiene in Asia, but its role in perianal disease is unknown.

**Purpose:**

To critically analyze and systematically review the current evidence regarding the effect of habitual bidet use on symptoms of benign perianal disease. *Data Sources.* A database search was conducted on MEDLINE and Epub Ahead of Print, Embase, ClinicalTrials.gov, the Cochrane Library, and ProQuest Dissertations. All studies on bidet use in pruritus ani, hemorrhoids, or anal fissures were included. *Data Extraction.* The studies were screened and critically analyzed by two independent reviewers in line with PRISMA guidelines.

**Results:**

Two prospective trials and 1 cross-sectional study found that habitual use of bidets had no impact on the odds of developing hemorrhoids or hemorrhoidal symptoms. One RCT concluded that using bidets was non-inferior to sitz bath for post-hemorrhoidectomy pain. Two prospective trials and 1 cross-sectional study determined that habitual bidet use may increase the odds of developing pruritus ani. Two case series found that habitual bidet use may cause perianal burns or anterior anal fissures. A meta-analysis was not performed because only a limited number of studies were available, and they were of variable quality.

**Conclusion:**

The current evidence does not identify using bidets as a treatment modality for perianal disease, and further research is warranted to study this increasingly utilized technology.

## 1. Introduction

Benign perianal disease includes a spectrum of conditions like hemorrhoids, fissures, fistulas, and ulcers [1]. These diseases carry significant morbidity, and symptoms including pain, bleeding, pruritus, discharge, difficulty with hygiene, and psychological distress [2]. Depending on the disorder, there is a plethora of treatment modalities that range from conservative, or nonoperative management to surgical interventions [3, 4].

Pain and bleeding are the most common symptoms of benign perianal disease, and a major component of disease morbidity [5]. Symptom relief may be best achieved with warm sitz baths, even when compared to topical anesthetics [4]. However, the frequent use of sitz baths can be inconvenient, leading to decreased compliance. This has stimulated interest in further water-based treatment modalities, such as a bidet, that could relieve symptoms of hemorrhoids and anal fissures in an accessible, efficient, and cost-effective manner.

A bidet is a sanitary toilet device used to clean the anal or urogenital area after urination, defecation, or menstruation. A water jet is sprayed on the anogenital region, which allows for easy rinsing and cleaning and potentially prevents localized trauma from wiping with toilet paper. During the COVID-19 pandemic, bidet sales increased substantially in North America [6]. Despite its novelty in North America, in most Asian and Middle Eastern countries, bidet use is the predominant form of perianal hygiene [7]. It is so common that in 2019 politicians in Thailand were publicly outraged when the parliament building was not equipped with bidets [8]. Bidets have been shown to improve the overall toileting comfort and cleanliness [9]. The physiological effects of sitz baths on perianal disorders can theoretically be reproduced with a bidet. Therefore, the objective of this study was to critically analyze and systematically review the current evidence regarding the effect of habitual bidet use on symptoms of benign perianal disease.

## 2. Materials and Methods

### 2.1. Search Strategy

**Figure 1:**
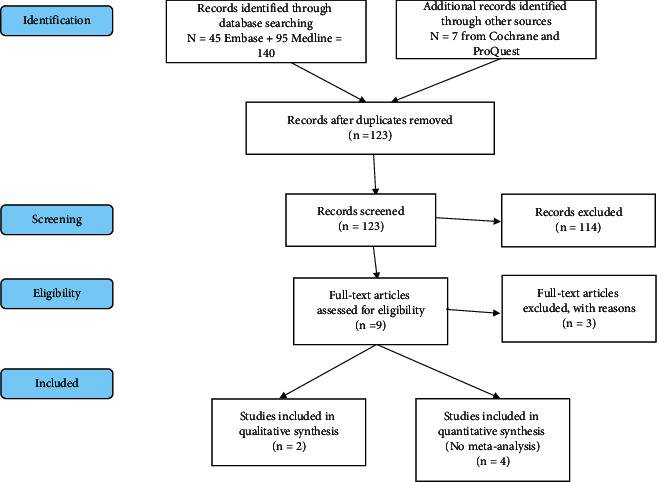
Preferred Reporting Items for Systematic Reviews and Meta-analyses flow chart [10].

A systematic review was performed according to the Preferred Reporting Items for Systematic Reviews and Meta-analyses checklist (Figure 1) [10]. This study has been registered in the International Prospective Register of Systematic Reviews, and the registration no. is CDR42021234392 [11]. Prior to conducting the review, the Cochrane Library, ClinicalTrials.gov, MEDLINE, Embase, and PubMed were searched, and no available systematic reviews on this topic were found.

With the assistance of a librarian, mesh terms from PubMed were identified by searching for “bidet,” “Japanese toilet,” “perianal,” “fissure,” “pruritus,” and “hemorrhoids.” The mesh terms were used to search in MEDLINE and Epub Ahead of Print (1946 to 2021), Embase (1946–2021), the Cochrane Library (https://www.cochranelibrary.com/), and ProQuest Dissertations (https://www.proquest.com/) to identify any published or unpublished studies on January 15th, 2021. A copy of the search strategy is provided in the Supplementary Materials (Appendix 2).

### 2.2. Study Selection and Quality Assessment

The studies were independently screened by 2 senior general surgery residents for inclusion and exclusion. There was a strong correlation of agreement between the two reviewers. Kappa Score = 0.74 (95.9% agreement). Disagreements were resolved through discussion with a third reviewer. Selected articles were then reviewed in detail for data extraction and quality appraisal. They were evaluated using critical appraisal instruments from Joanna Briggs Institute Meta-Analysis of Statistics Assessment and Review Instrument (JBI-MAStARI) (Appendix 1) [12].

### 2.3. Criteria for Inclusion and Exclusion

All studies on the use of bidets in hemorrhoids or anal fissures were included. This included publications in languages other than English. The review included randomized controlled trials (RCTs), observational studies, case series, case reports, conference abstracts, and theses and dissertations available online. Participants of any age or gender who used a bidet were included. Patients with new or previous diagnoses of symptomatic hemorrhoids or anal fissures were included. The symptoms included pruritus, pain, bleeding, discharge, perianal hygiene, psychological distress, and overall quality of life.

Review articles, advertisement posters, and news articles were excluded. Patients with diagnoses of urogenital diseases, such as vulvovaginitis, urinary tract infection, and lichen planus, were excluded. Patients with inflammatory bowel disease, malignancy, psoriasis, and other chronic dermatological conditions were also excluded.

### 2.4. Types of Intervention

The intervention of interest was the habitual use of bidet with or without the combination of other treatment modalities. The protocol for bidet use was not restricted and included variability in daily use, water temperature, water pressure, and width of water stream. Additional modalities for perianal hygiene such as use of toilet paper and sitz bath were not standardized. The review included studies with and without comparator groups. The comparator groups included either no bidet use, non-habitual bidet use, or habitual use of a sitz bath. Habitual use was defined as bidet use at least once/day.

### 2.5. Measured Outcomes

The primary outcome was the effect of habitual bidet use on the following:

1. The odds of developing new hemorrhoids.
2. The odds of developing symptoms of hemorrhoids such as bleeding, pain, pruritus, discharge, and psychological distress.
3. The odds of hemorrhoidal symptom resolution as measured on a self-administered questionnaire or physician assessment.

1. The odds of developing anal fissures.
2. The odds of developing symptoms of anal fissure.
3. The odds of symptom resolution in anal fissures.

### 2.6. Data Extraction

The data was extracted from the studies using Microsoft Excel®. Quantitative data containing odds ratios were compared to one another and presented in table format. Qualitative data were presented in a narrative form. A meta-analysis was not performed because of the heterogeneous nature of the studies and variations in their measured outcomes.

## 3. Results

### 3.1. Studies Yielded

The search identified 140 studies from Embase, MEDLINE, and Epub. A review of the retrieved studies found 18 duplicate results, resulting in 122 studies for initial review. Seven additional studies were identified from the Cochrane Library (https://www.cochranelibrary.com) and from ProQuest (https://www.proquest.com). From these 7 studies, 1 was duplicated in the previous search, 1 did not have any published results, and 3 did not pertain to bidet use, leaving only 1 additional study to be included. Therefore, a total of 123 abstracts (122 + 1) were then imported into Zotero® where two reviewers individually screened articles using the previously established inclusion and exclusion criteria (Figure 1).

Of the 123 abstracts, 114 were excluded from the review as they were incongruent with the inclusion criteria. Nine complete studies were then retrieved for detailed examination. From these 9 articles, 1 study was removed because the intervention was incorrectly identified as a bidet. Two additional studies were removed after discussion with a third reviewer because the outcomes did not specifically measure symptoms of perianal disease but instead used anal manometry pressures as a surrogate for symptoms. Therefore, only 6 studies met the previously established inclusion and exclusion requirements for data extraction. Four out of 6 studies were used to extract quantitative data for the results. The remaining 2 studies were discussed separately for their qualitative findings.

### 3.2. Characteristics of the Selected Studies

Of the 6 selected studies (Table 1), only 1 was a randomized controlled trial on the role of bidets in controlling symptoms of perianal pain, and it only had an abstract available online. Two studies were prospective cohorts that were conducted through an online questionnaire. Both of these studies measured the incidence of perianal symptoms with habitual bidet use at different time points. One additional study was a cross-sectional survey that also measured the correlation between bidet use and perianal symptoms. One study was a case series that correlated anterior anal fissures with bidet use. One study was a case report that linked bidet use to perianal burns. The case series and case report were used to report on qualitative findings from this review. There were significant variations in the study size, quality, design, methodology, and objectives between all 6 studies (Table 1).

### 3.3. Characteristics of Patients

In the 6 studies, the geographic settings were India, Japan, and South Korea. There was significant variation in participant numbers between the 6 studies ranging from 7,637 in 2 of the prospective cohorts to 1 participant in the case report. There were a total of 20,322 participants in all 6 studies. None of the studies identified a minimum number of participants required to meet statistical significance at 5%.

### 3.4. Interventions and Outcomes

In the RCT, Kwon et al. measured outcomes using a visualized analog score and quality of life assessment [13]. The 2 prospective trials by Kiuchi et al. and Asakura et al. had primary outcomes that were clearly identified as odds of developing hemorrhoids or symptoms of hemorrhoids after habitual bidet use [14, 15]. The cross-sectional survey by Tsunado et al. correlated bidet use with the odds of experiencing symptoms of hemorrhoids [16]. The case series by Garg described the development of anterior anal fissures in 10 patients after bidet use, reporting the resolution of symptoms with discontinuation of bidet use [17]. Shulman et al. described a case report on the development of a third-degree perianal burn with bidet use [18]. Neither the case series nor the case report had quantitative outcomes.

### 3.5. Qualitative Assessment and Bias

Kwon et al. performed an RCT, for which only the abstract was available online, but the measured outcomes were still extractable and thus included in this review [13]. The control and intervention groups were randomly assigned, and the outcomes were assessed using a validated visual analog score. A complete appraisal could not be performed given the absence of a manuscript and study methodology.

**Figure 2:**
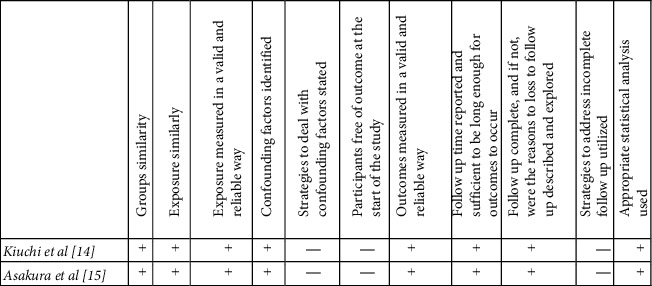
Cohort study quality assessment [12].

**Figure 3:**
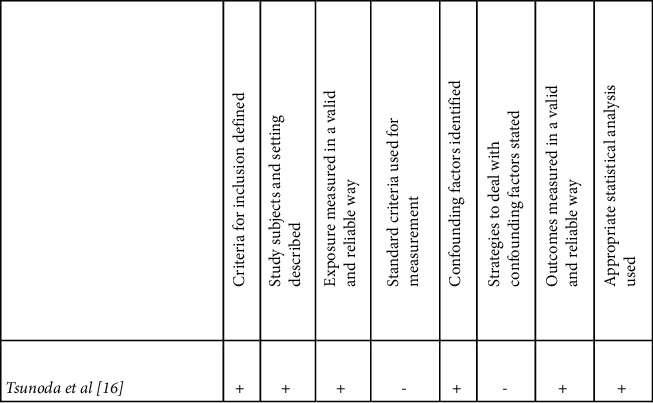
Cross-sectional survey quality assessment [12].

For prospective cohort studies (Figure 2), Kiuchi et al. and Asakura et al. are at risk of significant recall bias because the studies were conducted through a web-based survey at 1- and 3-year intervals [14, 15]. Furthermore, confounding factors were not controlled. For instance, findings of increased perianal pruritus in male patients after habitual bidet use could be attributed to multiple confounding factors such as age and bowel habits. The same biases and confounding factors are also found in the cross-sectional survey by Tsunoda et al. (Figure 3) [16]. All three studies had appropriate follow-up strategies and statistical analysis and scored similarly in the appraisal (Figures 2 and 3).

**Figure 4:**
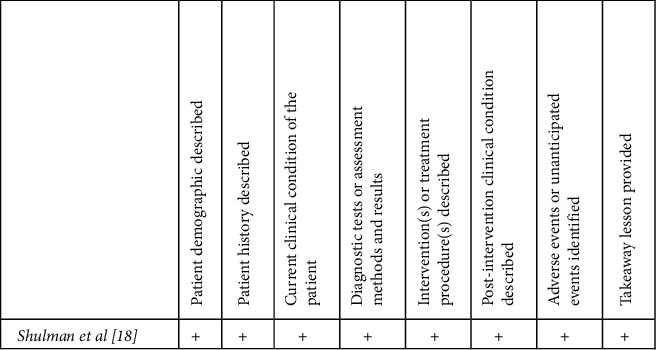
Case series quality assessment [12].

**Figure 5:**
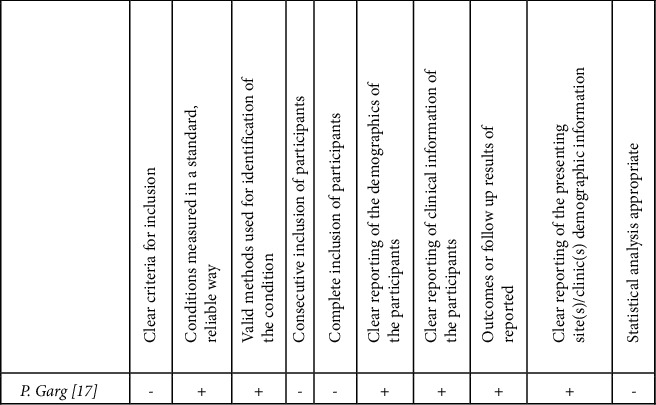
Case report quality assessment [12].

The case series by Garg (Figure 4) scored well in reporting the demographics of patients, disease development, and treatment provided [17]. However, a detailed description of the exposure to the bidet and possible confounding factors were not reported. The quality assessment of the case report by Shulman et al. revealed adequate and detailed description of a third-degree burn sustained in a patient suffering from multiple sclerosis after hot water bidet use (Figure 5).

### 3.6. Pruritus Ani and Bidet Use

The development of perianal pruritus with bidet use was assessed by three separate studies (Table 2). Two of the studies were prospective cohorts comparing results based on 1-year and 3-year follow-up [14, 15]. The third study was a cross-sectional survey [16]. Kiuchi et al. compared the habitual use of bidet (>once/day) with non-habitual use (<once/week) and reported an odds ratio of 1.267 (95% CI: 0.958–1.1682) in males and 1.021 (95% CI: 0.80–1.345) in females for developing pruritus with habitual bidet use after one year [14]. Asakura et al. followed the same prospective cohort after 3 years and reported an odds ratio of 1.36 (95% CI: 1.067–2.275) in males and 1.14 (95% CI: 0.89–1.46) in females for developing pruritus with habitual bidet use [15]. Between the two studies, only the odds of developing pruritis in males with habitual bidet use after 3 years were statistically significant.

Tsunoda et al. used a cross-sectional survey to compare the habitual use of bidet (≥twice/day) with non-habitual use (≤once/day) and reported an odds ratio of 2.68 (95% CI: 1.15–2.36) for developing pruritus in males after habitual bidet use, indicating statistical significance. The odds of developing pruritis in females were not reported as a function of frequency of bidet use. The same study also found that a large number of participants used the bidet to aid in defecation and that men >50 years of age used bidets most frequently [16].

### 3.7. Hemorrhoids and Bidet Use

Kiuchi et al. focused on multiple aspects of bidet use and anorectal symptoms including the odds of developing hemorrhoids with habitual bidet use (>once/day) [14]. The odds ratio of developing hemorrhoids diagnosed by a physician was 1.013 (95% CI: 0.49–2.1) in males and 1.923 (95% CI: 0.950–3.9) in females. Furthermore, they reported the odds of developing subjective symptoms of hemorrhoids at an odds ratio of 1.117 (95% CI: 0.799–1.5) for males and 1.058 (95% CI: 0.746–1.5) for females, neither of which is statistically significant.

In the prospective cohort study by Asakura et al., they reported an odds ratio of 1.67 (95% CI: 0.90–3.10) for the development of hemorrhoids in males and 1.12 (95% CI: 0.62–2.03) in females after 1 year [15]. The odds ratio of developing subjective symptoms of hemorrhoids was 1.18 (95% CI: 0.86–1.61) in males and 1.130 (95% CI: 0.78–1.62) in females. Thus, neither of the studies reported any significant risk of developing hemorrhoids or hemorrhoidal symptoms at the 1- or 3-year follow-up (Table 3).

### 3.8. Bidet versus Sitz Baths for Pain

The RCT of Kwon et al. enrolled patients undergoing hemorrhoidectomy who were assigned to either an electronic bidet group or a conventional sitz bath to assess pain control and wound healing after surgery. The treatment had to be applied at least once a day. Pain scores were assessed on a visual analog scale (VAS) (range: 0–100) for 4 weeks before and after the use of electronic bidet or conventional sitz bath. Wound healing status, patient satisfaction, and convenience were also assessed. 75 patients were enrolled. 41 were assigned to the conventional sitz bath group and 34 to the electronic bidet group. Postoperative pain at 1 week was similar between the two groups with VAS score of 38.26 ± 21.87 in the electronic bidet group and 40.71 ± 21.64 in the conventional sitz bath group [13]. There was no significant difference between the two groups in satisfaction or wound epithelialization, and the authors concluded that a bidet can be used as an alternative to sitz baths.

### 3.9. Anal Fissures and Bidet Use

Garg reported a case series on 1 female and 9 male patients who presented with an anterior fissure and a history of bidet use [17]. Two out of ten patients had acute fissures while 8/10 had chronic fissures. Chronicity of the fissure was defined as having either fibrosis at the base of the fissure or a sentinel pile distal to the fissure. All patients were asked to stop using the bidet and were simultaneously initiated on a standardized treatment protocol which included local analgesics, stool softeners, sitz baths, and nitroglycerin ointments. Nine of 10 patients had complete resolution of symptoms within 3 weeks while 1 patient did not respond to the treatment and required surgical intervention. The report concluded that bidet use was the cause of the anterior fissures and abstaining from bidet use resulted in resolution of symptoms. However, causation cannot be ascribed to the bidet use, nor can resolution be attributed to cessation of use given the concurrent application of other validated treatments including topical nitroglycerin.

### 3.10. Risk Factors for Complications in Bidet Users

Shulman et al. presented a case of a 69-year-old female with a third-degree burn in the right perineal region after bidet use [18]. The patient was known to have multiple sclerosis and had received high dose steroids 6 weeks prior to presentation due to exacerbation of her underlying disease. The burn was debrided and treated with oral and topical antimicrobial treatments. The main conclusion was that patients with peripheral neuropathy and reduced sensation need to be warned against burns if using warm water bidets.

## 4. Discussion

Benign perianal diseases represent one of the most common referrals to colorectal and general surgeons [19]. In this review, the focus was on hemorrhoids and anal fissures, which have symptoms directly related to defecation practices. Approximately 70% of patients note bright-red blood after wiping with a toilet paper, and patients with fissures often report “passing razor-blades” [20]. Patients are routinely recommended to use sitz baths for symptom control after defecation, along with healthy toileting habits and fiber, with up to 50% reporting symptom resolution [21]. The exact mechanism by which sitz baths work remains unclear. The most common hypothesis is that warm sitz baths relieve pain by relaxing the internal anal sphincter, which decreases the rectal neck pressure and the internal anal sphincter activity [22]. Although the exact mechanism of sitz baths is not established, it is a major component of conservative management for both hemorrhoids and fissures [3, 4, 23]. Therefore, we considered whether using a continuous stream of water after defecation, in the form of a bidet, offers a convenient alternative.

There were 6 studies included in the review with a variety of study designs including prospective cohort, randomized control trial, cross-sectional design, case series, and a case report. Due to the variability in study designs, a meta-analysis was not performed; however, the overall findings shed light on the role of bidets in perianal disease.

The only statistically significant finding in this review was the correlation between bidet users and pruritus. Kiuchi et al. demonstrated no significant increase in the incidence of anal pruritis after one year of habitual bidet use. However, a three-year follow-up study was able to show a significant increase of anal pruritis in male bidet users with an odds ratio of 1.36 [14]. This finding was also supported by Tsunoda et al. who found that male bidet users had a high prevalence of pruritus with an odds ratio of 2.68 [16]. The findings in these studies can be attributed to multiple reasons. Reverse causation is a key factor as patients with perianal discomfort are more likely to use a bidet to alleviate those symptoms. Additionally, Asakura et al. reported that the majority of male patients with irritated perianal skin were young, suffered from constipation, and may have used the bidet to aid in defecation [15]. This finding is also recognized by Tsunoda et al. who reported that at least 30% of patients used the bidet prior to defecation with 70% expecting its aid in defecation [16]. Furthermore, younger individuals tend to be more physically active, which causes increased moisture in the perineum and contributes to pruritus [24]. One of the main attributable causes of pruritus ani is local irritation and is frequently secondary to leakage of fecal content [24, 25]. A future study that controls these confounding factors is more likely to establish causation between bidet use and pruritus.

A key finding from this review was that bidet use did not increase the risk of developing hemorrhoids or symptoms of hemorrhoids, after 1 or 3 years of use, demonstrating that implementing bidet use as a treatment does not exacerbate the disease [14, 15]. From a physiological point of view, bidet use should improve hemorrhoidal disease and anal fissures. Hemorrhoid development is multifactorial and is attributed to the abnormal dilation of the internal hemorrhoidal venous plexus and increased anal sphincter pressure [26]. Anal fissures are caused by a cycle of increased internal sphincter tone leading to constipation, and trauma from passage of hard stools, which further increases internal sphincter tone [27]. The resultant hypertonicity of the internal anal sphincter leads to ischemia of the posterior anal commissure, preventing healing [27]. Thus, reducing internal anal sphincter tone during evacuation should reduce the burden of hemorrhoids and anal fissures and promote their healing. In fact, Tsunoda et al. found that a large number of participants used the bidet as an aid prior to defecation [16].

An unexpected finding was the case series that identified 10 patients with anterior anal fissures that were attributed to bidet use. Given the study design, no comparator group was identified [17]. Therefore, direct causation between bidet use and fissure development cannot be deduced. The study also failed to elaborate on the frequency, pressure, temperature, width, and type (handheld/seated) of bidets used. These variations in bidet setting can have different effects, with colder temperatures and higher pressures resulting in higher sphincter tones, which can contribute to fissures [28, 29]. A 2011 study compared pressure at the anal pressures after 1 minute of bidet use using various bidet settings. There was a significant reduction in anal pressures when a warm (38°C), low to medium pressure (40–80 mN) bidet was used [28]. However, higher jet pressures (160–200 mN) increased anal pressures, even when warm water (38°C) was used. A follow-up investigation compared anal pressures after conventional sitz bath and bidet use after 3 minutes and found that both interventions had similar effects on reducing anal pressure (Table 4) [29]. These findings show the potential of using bidets as an alternative to sitz baths for anal fissures, but a specific pressure and temperature requirement might be required.

Although none of the studies investigated the role of bidets in pain secondary to fissures and hemorrhoids, Kwon et al. found that patients with postsurgical perianal pain responded well to bidet use. Conventional sitz baths and bidet use had similar VAS (visual analog score) values, and there was no significant difference in any of the measured outcomes [13].

Bidet use is safe and convenient, but precautions should be taken in certain individuals. One of the included studies was a case report of a third-degree perineal burn after hot water bidet use in a patient with multiple sclerosis on immunosuppressive therapy [18]. Therefore, avoiding prolonged use of bidets with hot water in patients with altered perianal sensation is crucial. This includes patients with diabetes, neurological disorders, and chemotherapy induced neuropathy [18].

Finally, bidets may offer a significant cost benefit. The 23 kg of toilet paper used by the average North American require 140 litres of water to produce, and an additional 6.1 litres to flush annually. Given the environmental and financial impact of toilet paper consumption and the fluctuating supply of toilet paper during the COVID-19 pandemic, bidets can offer an economic advantage [30]. This advantage is further realized when considering the millions of patients who may not require invasive procedures for perianal disease. Among these procedures, a triple-rubber band ligation is generally considered to be the most cost-effective and efficient method of controlling hemorrhoidal symptoms, which costs $50–80 for just the medical equipment [31, 32]. Many patients also require multiple banding procedures for symptom control [33]. This could potentially lessen the burden on the healthcare system, especially in the midst of this current pandemic.

Surprisingly, no trials assessed bidet use specifically for the treatment of established hemorrhoidal or fissure disease. The published studies assessed whether habitual bidet use was associated with development of either of these conditions, but no studies assessed use as therapy. Given the validated role of sitz baths in treatment of perianal disease and the mechanistic and physiological similarities to bidet use, further studies to assess therapeutic bidet use are warranted.

## 5. Conclusion

Bidets use may be an untapped alternative to sitz baths. The current literature provides no strong evidence to support or discourage the use of bidets in perianal disease. However, the findings indicate the potential of using bidets to treat hemorrhoids and anal fissures in an accessible, efficient, and cost-effective manner. Further research into this technology, which should include randomized controlled trials, is warranted.

## Figures and Tables

**Table 1:** Characteristics and design of each study.

Study	Study type	*N*	Methodology	Intervention	Comparator	Outcomes measured
Kiuchi et al. [14]	Prospective cohort	7,637	Web-based survey with 1-year follow-up	Habitual users of bidet (at least once/day)	Non-habitual users (used less than once/week)	Odds of developing hemorrhoids, symptoms of hemorrhoids, and pruritus

Asakura et al. [15]	Prospective cohort	7,637	Web-based survey with 3-year follow-up	Habitual users of bidet (at least once/day)	Non-habitual users (used less than once/week)	Odds of developing hemorrhoids, symptoms of hemorrhoids, and pruritus

Tsunoda et al. [16]	Cross-sectional survey	4,963	Web-based survey	Use of bidet twice or more a day	Use of bidet once/day or less	16 questions related to bidet use and perianal symptoms (Table 3, multivariate analyses of pruritus ani with bidet use)

Kwon et al. [13]	Randomized controlled trial	74	Post-hemorrhoidectomy patients were instructed to use a bidet or sitz bath for 4 weeks	Bidet use (at least once/day)	Conventional sitz bath	Pain (measured on a visual analog score)

Garg [17]	Case series	10	Patients presented with anterior fissures after bidet use	Discontinuation of bidet use	None	Resolution of fissure with discontinuation of bidet use

Shulman et al. [18]	Case report	1	3rd degree burn caused by use of a bidet in a patient with multiple sclerosis	Bidet use	None	Burn symptoms caused by bidet use

**Table 2:** Odds of developing pruritus ani with hemorrhoids after habitual bidet use.

Study	Study type	*N*	Comparison		Outcome extracted	Odds ratio of pruritus	
			Intervention group	Comparator group		M	F
Kiuchi et al. [14]	Prospective cohort	7,637	Using bidet at least once/day	Using bidet less than once/week	Odds of developing pruritus at 1 year	1.267 (0.958–1.681)	1.021 (0.80–1.345)
Asakura et al. [15]	Prospective cohort	7,637	Using bidet at least once/day	Using bidet less than once/week	Odds of developing pruritus at 3 years	1.36^*∗*^ (1.067–1.75)	1.14 (0.89–1.46)
Tsunoda et al. [16]	Cross-sectional survey	2,300	Using bidet twice or more a day	Using bidet less than twice a day	Correlation of pruritus ani with increased frequency of bidet use	1.68^*∗*^ (1.15–2.36)	N/A

The asterisk implies significance at *P* < 0.05.

**Table 3:** Odds of developing hemorrhoids and subjective hemorrhoidal symptoms after habitual bidet use.

Study	Comparison		Outcome	Odds of developing hemorrhoids (diagnosed by a physician)		Odds of developing subjective symptoms of hemorrhoids	
	Intervention group	Comparator group		M	F	M	F
Kiuchi et al. [14]	Using bidet at least once/day	Using bidet less than once/week	Odds of developing hemorrhoidal symptoms at 1 year	1.013 (0.489–2.106)	1.923 (0.950–3.971)	1.117 (0.799–1.562)	1.058 (0.746–1.508)

Asakura et al. [15]	Using bidet at least once/day	Using bidet less than once/week	Odds of developing hemorrhoidal symptoms at 3 years	1.67 (0.90–3.10)	1.12 (0.62–2.03)	1.18 (0.86–1.61)	1.13 (0.78–1.62)

**Table 4:** Effect of bidet use on resting anal pressure.

Study	Study type	*N*	Methodology	Comparator group	Intervention group	Outcome extracted	Anal pressure (mmHg)		
							Comparator	Intervention	Difference
Ryoo et al. [28]	Prospective cohort	20	20 healthy volunteers had anal pressures measured at the anal high-pressure zone (HPZ) before and after 1 minute of bidet use with different settings	Before bidet use	Bidets set at different pressures (40 mN and 80 mN), different temperatures (24°C and 38°C), and different jet types (wide or narrow)	Pressure at HPZ at 38°C, 40 mN, and wide jet	96.1 ± 22.5	81.9 ± 23.3	14.2^*∗*^ (*p* < 0.01)

Ryoo et al. [29]	Prospective cohort	40	40 healthy volunteers had anal pressures measured at the HPZ before and after 3 minutes of bidet use	Change in the anal HPZ after 3 minutes of sitz bath use	Change in the anal HPZ after 3 minutes of bidet use	Change in pressure at HPZ after sitz bath and bidet use	88.1 − 69.6 = 18.5^*∗*^ (*p* < 0.01)	90.2 − 71.3 = 18.9^*∗*^ (*p* = 0.990)	0.04 (*p* = 0.990)

## Data Availability

The search strategy used to support the findings of this study is included within the Supplementary Materials.

## Supplementary Materials

Supplementary Materials Appendix 1: JBI critical appraisal tool. This is a copy of the Joanna Briggs Institute Critical Appraisal Evaluation form used to critically appraise research studies that are included in a systematic review. The evaluation tool is published by the institute and validated in the role of critically appraising articles for systematic reviews [12]. We used this tool to evaluate the studies in this systematic review. Appendix 2: database search strategy. This document describes the search strategy used to obtain the findings from both PubMed and MEDLINE to obtain the articles we found for the systematic review. Appendix 3: completed PRISMA checklist. The Preferred Reporting Items for Systematic Reviews and Meta-analyses (PRISMA) is a validated checklist used to improve transparency in systematic reviews [10].
