# Supplementary material for: Be Kind to Your Behind: A Systematic Review of the Habitual Use of Bidets in Benign Perianal Disease
Source: Evid Based Complement Alternat Med. 2022 May 31;2022:1633965. doi: 10.1155/2022/1633965 (PMC9173983; doi:10.1155/2022/1633965)
Supplement: Supplementary Materials — Appendix 1: JBI critical appraisal tool. This is a copy of the Joanna Briggs Institute Critical Appraisal Evaluation form used to critically appraise research studies that are included in a systematic review. The evaluation tool is published by the institute and validated in the role of critically appraising articles for systematic reviews [12]. We used this tool to evaluate the studies in this systematic review. Appendix 2: database search strategy. This document describes the search strategy used to obtain the findings from both PubMed and MEDLINE to obtain the articles we found for the systematic review. Appendix 3: completed PRISMA checklist. The Preferred Reporting Items for Systematic Reviews and Meta-analyses (PRISMA) is a validated checklist used to improve transparency in systematic reviews [10]. [file 1633965.f1.zip › 163396.f1/Appendix 1. JBI Critical Appraisal Tool.docx]

**APPENDIX. 1**

JBI Critical Appraisal Checklist for cohort studies [13]

Reviewer ______________________________________ Date_______________________________

Author_______________________________________ Year_________ Record Number_________

|  | Yes | No | Unclear | Not applicable |
| --- | --- | --- | --- | --- |
| 1. Were the two groups similar and recruited from the same population? | □ | □ | □ | □ |
| 1. Were the exposures measured similarly to assign people 2. to both exposed and unexposed groups? | □ | □ | □ | □ |
| 1. Was the exposure measured in a valid and reliable way? | □ | □ | □ | □ |
| 1. Were confounding factors identified? | □ | □ | □ | □ |
| 1. Were strategies to deal with confounding factors stated? | □ | □ | □ | □ |
| 1. Were the groups/participants free of the outcome at the start of the study (or at the moment of exposure)? | □ | □ | □ | □ |
| 1. Were the outcomes measured in a valid and reliable way? | □ | □ | □ | □ |
| 1. Was the follow up time reported and sufficient to be long enough for outcomes to occur? | □ | □ | □ | □ |
| 1. Was follow up complete, and if not, were the reasons to loss to follow up described and explored? | □ | □ | □ | □ |
| 1. Were strategies to address incomplete follow up utilized? | □ | □ | □ | □ |
| 1. Was appropriate statistical analysis used? | □ | □ | □ | □ |

Overall appraisal: Include □ Exclude □ Seek further info □
