# Supplementary material for: Be Kind to Your Behind: A Systematic Review of the Habitual Use of Bidets in Benign Perianal Disease
Source: Evid Based Complement Alternat Med. 2022 May 31;2022:1633965. doi: 10.1155/2022/1633965 (PMC9173983; doi:10.1155/2022/1633965)
Supplement: Supplementary Materials — Appendix 1: JBI critical appraisal tool. This is a copy of the Joanna Briggs Institute Critical Appraisal Evaluation form used to critically appraise research studies that are included in a systematic review. The evaluation tool is published by the institute and validated in the role of critically appraising articles for systematic reviews [12]. We used this tool to evaluate the studies in this systematic review. Appendix 2: database search strategy. This document describes the search strategy used to obtain the findings from both PubMed and MEDLINE to obtain the articles we found for the systematic review. Appendix 3: completed PRISMA checklist. The Preferred Reporting Items for Systematic Reviews and Meta-analyses (PRISMA) is a validated checklist used to improve transparency in systematic reviews [10]. [file 1633965.f1.zip › 163396.f1/Appendix 2. Database search strategy .docx]

**APPENDIX 2**

**Database search strategy for Medline and ePub**

1. bidet?.mp.

2. ((automated or japanese) adj3 toilet?).mp. [mp=title, abstract, original title, name of substance word, subject heading word, floating sub-heading word, keyword heading word, organism supplementary concept word, protocol supplementary concept word, rare disease supplementary concept word, unique identifier, synonyms]

3. ((cleaning or wash* or (wash and dry) or electronic) adj3 toilet?).mp. [mp=title, abstract, original title, name of substance word, subject heading word, floating sub-heading word, keyword heading word, organism supplementary concept word, protocol supplementary concept word, rare disease supplementary concept word, unique identifier, synonyms]

4. (shattaf? or shataf?).mp. [mp=title, abstract, original title, name of substance word, subject heading word, floating sub-heading word, keyword heading word, organism supplementary concept word, protocol supplementary concept word, rare disease supplementary concept word, unique identifier, synonyms]

5. technology-assisted toilet?.mp.

6. muslim shower?.mp.

7. power jet?.mp.

8. (spray* adj3 (anal or anus)).mp. [mp=title, abstract, original title, name of substance word, subject heading word, floating sub-heading word, keyword heading word, organism supplementary concept word, protocol supplementary concept word, rare disease supplementary concept word, unique identifier, synonyms]

9. (lavage adj3 toilet?).mp. [mp=title, abstract, original title, name of substance word, subject heading word, floating sub-heading word, keyword heading word, organism supplementary concept word, protocol supplementary concept word, rare disease supplementary concept word, unique identifier, synonyms]

10. Bathroom Equipment/

11. Toilet Facilities/

12. or/1-11

13. (anorectal or anorectum or anal or anus or ano or ani).mp. [mp=title, abstract, original title, name of substance word, subject heading word, floating sub-heading word, keyword heading word, organism supplementary concept word, protocol supplementary concept word, rare disease supplementary concept word, unique identifier, synonyms]

14. (perianal or peri-anal).mp. [mp=title, abstract, original title, name of substance word, subject heading word, floating sub-heading word, keyword heading word, organism supplementary concept word, protocol supplementary concept word, rare disease supplementary concept word, unique identifier, synonyms]

15. fissure?.mp.

16. pruritus.mp.

17. (hemorrhoid? or haemorrhoid? or hemorrhoidal or haemorrhoidal).mp. [mp=title, abstract, original title, name of substance word, subject heading word, floating sub-heading word, keyword heading word, organism supplementary concept word, protocol supplementary concept word, rare disease supplementary concept word, unique identifier, synonyms]

18. or/13-17

19. 12 and 18
